# Mechanistic Pathways of Malignancy in Breast Cancer Stem Cells

**DOI:** 10.3389/fonc.2020.00452

**Published:** 2020-04-30

**Authors:** Saghar Yousefnia, Farzad Seyed Forootan, Shiva Seyed Forootan, Mohammad Hossein Nasr Esfahani, Ali Osmay Gure, Kamran Ghaedi

**Affiliations:** ^1^Department of Cell and Molecular Biology and Microbiology, Faculty of Biological Science and Technology, University of Isfahan, Isfahan, Iran; ^2^Department of Cellular Biotechnology at Cell Science Research Center, Royan Institute of Biotechnology, ACECR, Isfahan, Iran; ^3^Legal Medicine Research Center, Legal Medicine Organization, Tehran, Iran; ^4^Department of Molecular and Clinical Pharmacology, MRC Centre for Drug Safety Science, Institute of Translational Medicine, University of Liverpool, Liverpool, United Kingdom; ^5^Department of Molecular Biology and Genetics, Faculty of Science, Bilkent University, Ankara, Turkey

**Keywords:** angiogenesis, breast cancer stem cell, chemotherapy and radiotherapy resistance, invasion, metastasis

## Abstract

Breast cancer stem cells (BCSCs) are the minor population of breast cancer (BC) cells that exhibit several phenotypes such as migration, invasion, self-renewal, and chemotherapy as well as radiotherapy resistance. Recently, BCSCs have been more considerable due to their capacity for recurrence of tumors after treatment. Recognition of signaling pathways and molecular mechanisms involved in stemness phenotypes of BCSCs could be effective for discovering novel treatment strategies to target BCSCs. This review introduces BCSC markers, their roles in stemness phenotypes, and the dysregulated signaling pathways involved in BCSCs such as mitogen-activated protein (MAP) kinase, PI3K/Akt/nuclear factor kappa B (NFκB), TGF-β, hedgehog (Hh), Notch, Wnt/β-catenin, and Hippo pathway. In addition, this review presents recently discovered molecular mechanisms implicated in chemotherapy and radiotherapy resistance, migration, metastasis, and angiogenesis of BCSCs. Finally, we reviewed the role of microRNAs (miRNAs) in BCSCs as well as several other therapeutic strategies such as herbal medicine, biological agents, anti-inflammatory drugs, monoclonal antibodies, nanoparticles, and microRNAs, which have been more considerable in the last decades.

## Introduction

The most common cancer among women all over the world is breast cancer (BC). The prevalence of BC is increasing; according to the recent report (2020), 279,100 new cases and 42,690 deaths of BC have been estimated in the United States (https://doi.org/10.3322/caac.21590). Approximately 25% of cancers in women and 15% of cancer deaths are caused by BC. About 0.8–1% of all BCs are due to male breast carcinoma, although the incidence of BC in men has increased recently. It has been estimated that the prevalence of BC in the world will reach 2.3 million by 2030 (1, 2). The most important factors for promoting BC are high expression level of estrogen, estrogen receptor (ER), progesterone receptor (PR), and human epidermal growth factor receptor 2 (HER2). On the other hand, mutation in tumor suppressor genes such as BRCA1 and BRCA2 is the most important reason for familial BC (3). There are two types (ductal and lobular) and multiple subtypes [luminal A and B, basal-like triple negative breast cancer (TNBC), HER2+, and claudin-low] of BC that are classified by molecular and histological phenotypes. Each type and subtype of BC are categorized to non-invasive and invasive (2, 4–7). A population of cancer cells in BC tissues makes the treatment of BC obscure. Cancer stem cells (CSCs) are a type of BC cells with some stemness phenotypes such as self-renewal, differentiation, metastasis, migration, and therapeutic resistance that make tumors more progressive and aggressive (8–10). Breast tumor consists of 2% breast cancer stem cells (BCSCs), in which their resistance to chemotherapy and radiotherapy can cause treatment failure and disease recurrence (8). There is a controversy among researchers about the origin of BCSCs, in which normal stem cells, progenitor cells, or differentiated cells can be considered as an origin of BCSCs (11). Deregulation of signaling pathways including mitogen-activated protein (MAP) kinase, PI3K/Akt/nuclear factor kappa B (NFκB), TGF-β, hedgehog (Hh), Notch, Wnt/β-catenin, and Hippo pathway in normal stem cells, progenitor cells, or differentiated cells may transform them to CSCs due to genetic and epigenetic changes (12–14). In addition, the expression of the specific molecules in these signaling pathways can be deregulated in BCSCs as well as microRNAs (miRNAs). It has been reported that there are several miRNAs that have been upregulated in BCSCs (Table 1). Downregulation of these miRNAs has been suggested as a novel treatment strategy of BC (Table 2). Alternative strategies are being used to target BCSCs directly. In this review, we have summarized the malignant characterization of BCSCs such as drug and radiotherapy resistance, metastasis and angiogenesis, biomarkers, and mechanisms involved in drug and radiotherapy resistance. We have assessed signaling pathways that are responsible for maintaining stemness phenotypes as well as miRNAs in BCSC. The recognition of signaling pathways and molecular mechanisms involved in stemness phenotypes of BCSCs would be effective for discovering novel treatment strategies to target BCSCs. Finally, we have reviewed several therapeutic strategies that have been investigated in the last decades.

**Table 1:** MicroRNAs (miRNAs) upregulated in breast cancer stem cells (BCSCs).

**miRNA**	**Target**	**Result**	**References**
miR-10b	Hox D10	-Metastasis	(15)
miR-21	PTEN	-Activates Akt/ERK1/2 pathways -EMT -Invasion -Metastasis	(16, 17)
miR-22	Hypermethylation of miR-200 promoter, miR-200 inactivation, ZEB1/2, and BMI1 expression	-EMT -Metastasis	(18)
miR-125	Bak1	Promotes CSC maintenance	(19)
miR-181	BRCA1	Promotes CSCs phenotypes	(20)
miR-221/222	PTEN	-Activate PI3K/Akt pathway -xIncrease proliferation	(21)
	Akt phosphorylation		

*Hox D10, Homebox protein D10; PTEN, phosphatase and tensin homolog; EMT, epithelial–mesenchymal transition; ZEB1/2, zinc finger E-box binding homeobox 1/2; BMI1, B lymphoma Mo-MLV insertion region 1; homolog, a member of the polycomb repression complex 1 family; Bak1, BCL2 antagonist/killer 1; BRCA1, breast cancer type 1 susceptibility protein*.

**Table 2:** MicroRNAs (miRNAs) targeting breast cancer stem cells (BCSCs).

**miRNA**	**Target**	**Result on BCSCs**	**References**
miR-7	KLF4	-Reduces stemness phenotypes -Inhibits pluripotent potential of stem cells	(22)
miR-9	Notch signaling	Reduces metastasis	(23)
miR-16	WIP1	-Reduces self-renewal -Increases sensitivity to doxorubicin (Dox)	(24)
miR-23b	MARCKS	-Inhibiting cell cycle -Inhibiting motility	(25)
miR-29b	-SPIN1 -Wnt/β-catenin and Akt signal pathways -VEGFA -PDGFA/B/C -MMP2/9, ITGA6, -ITGB1, TGFβ2/3	-Inhibits self-renewal and growth -Inhibits invasion and metastasis	(26)
miR-30a	Protein AVEN	-Inhibits the growth -Induces apoptosis	(27)
miR-30e	-Ubc9 -ITGB3	-Inhibits self-renewal -Induces apoptosis	(28)
miR-34 family (miR-34a and miR-34c)	-Notch signaling -Notch4	-Reduces cancer stem cell phenotypes -Suppresses EMT -Suppresses metastasis -Increases sensitivity to Dox and paclitaxel	(23, 29, 30)
miR-93	Sox4	-Reduces stemness phenotypes -Promotes differentiation -Inhibits pluripotent potential of stem cells	(31)
miR-126/miR-206/miR-335	-Sox4 -Tenascin C	-Reduces stemness phenotypes and proliferation -Inhibits metastasis and migration	(32)
miR-128	-Nanog -Snail	-Reduces stemness phenotypes -Inhibits pluripotent potential of stem cells	(33, 34)
miR-140	-Sox9 -ALDH1	-Reduces stemness phenotypes -Inhibits pluripotent potential of stem cells	(35)
miR-148	-BMI1 -ABCC5	-Inhibits progression -Induces apoptosis -Increases sensitivity to Dox	(33, 34)
miR-153	HIF1α	Inhibits angiogenesis	(36)
miR-200 family (miR-200a, miR-200b, and miR-200c)	-BMI1 -Suz12 -Notch pathway components, Jagged1, Maml2/3 -ZEB1/2	-Suppresses colony formation -Suppresses tumor formation -Suppresses invasion -Suppresses EMT	(37–39)
miR-600	-SCD1 enzyme -Wnt/β-catenin pathways	Promotes differentiation	(40)
miR-708	Neuronatin ERK/FAK pathway	Inhibits migration and metastasis	(41)
let-7	-H-RAS -MYC -HMGA2 -IL-6 -ERα	-Inhibits self-renewal -Inhibits pluripotent potential of stem cells	(42, 43)

*KLF4, Kruppel-like factor 4; WIP1, wild-type p53-induced phosphatase 1; MARCKS, myristoylated alanine rich protein kinase C substrate; SPIN1, Spindlin 1; VEGFA, vascular endothelial growth factor A; PDGFA/B/C, platelet-derived growth factor A/B/C; MMP2/9, matrix metalloproteinases 2/9; ITGA6, integrin subunit alpha 6; ITGB1, integrin subunit beta 1; TGFβ2/3, transforming growth factor beta 2/3; Protein AVEN, apoptosis and caspase activation inhibitor; Ubc9, hypothetical protein; ITGB3, integrin subunit beta 3; EMT, epithelial–mesenchymal transition; ALDH1, aldehyde dehydrogenase 1; BMI1, B lymphoma Mo-MLV insertion region 1 homolog, a member of the polycomb repression complex 1 family; ABCC5, multidrug resistance-associated protein 5; HIF1α, hypoxia-inducible factor 1α; Suz12, polycomb repressive complex 2 subunit; Maml2/3, mastermind-like Notch coactivators 2/3; ZEB1/2, zinc finger E-box binding homeobox 1; SCD1, stearoyl-CoA desaturase-1; HMGA2, high-mobility group AT-hook 2; IL-6, interleukin 6; ERα, estrogen receptor α*.

## BCSC Markers

BCSCs express variety of specific markers such as CD44^+^/CD24^−^, CD326 (EpCAM), epithelial specific antigen (ESA), and aldehyde dehydrogenase (ALDH) activity in different types of BC. Unlike CD24, known as a differentiated BC cells marker, CD44 has been identified as a stemness marker on the surface of BCSCs. CD44 is bonded to hyaluronic acid (HA) and extracellular matrix proteins such as osteopontin (OPN) and matrix metalloprotease (MMP) (44). CD44 with receptor tyrosine kinase (RTK) may control cell adhesion, migration, and transmission of proliferation signals in BCSCs (45). Several signaling pathways such as Rho GTPases, Ras-MAPK, and PI3K/Akt involved in the regulation of cell adhesion, migration, invasion, and epithelial–mesenchymal transition (EMT) are mediated by CD44 (46). However, CD44^+^ cannot be very helpful as a definite marker for isolation and detection of BCSCs in all types of BC (5, 47, 48). Luminal and basal/mesenchymal BCs have CD44^−^/CD24^+^ and CD44^+^/CD24^−^ phenotypes, respectively, whereas basal/epithelial BC is positive for both markers (49). Frequency of BCSCs with CD44^+^/CD24^−^ is significantly more in TNBCs (44). In addition, PR and ER messenger RNA (mRNA) levels in cells with the markers of CD44^+^/CD24^−^/ALDH^+^ significantly are less in comparison with cells with phenotype of CD44^−^/CD24^+^ (11). ALDH activity is a better predictive marker in parallel with higher tumorigenic activity *in vivo* in comparison with CD44/CD24 markers (50, 51). ALDH enzyme is responsible for intracellular aldehyde oxidation and has a critical role in differentiation of stem cells (52). To detect ALDH activity using Aldeflour assay kit, ALDH converts BODIPY-aminoacetaldehyde substrate to BODIPY-aminoacetate, a fluorescent product detectable by flow cytometry (51). The other important marker is ESA or CD326. ESA is a protein marker that is expressed on the surface of BCSCs essential for cell adhesion, proliferation, migration, and invasion of BC cells through Wnt signaling pathway (53). A regulated intramembrane proteolysis by ADAM metallopeptidase domain 17 (ADAM17) and Presenilin-2 (PSEN2) involves breakage of EpCAM intracellular domain(EpICD). EpICD binds to a half LIM domains 2 (FHL2) and β-catenin and forms a nuclear protein complex, which expresses genes involved in stemness physiological features (54). The other markers mostly used for isolation and identification of BCSCs in all types of BCs are CD133, CD166, Lgr5, CD47, and ABCG2 (55). A recent study indicated that transglutaminase (TG2) is expressed highly in CSCs and is involved in the expression of CSC markers, proliferation, drug resistance, migration, invasion, and EMT of CSCs. This protein is dependent to Ca^2+^ and GTP localized in cytosol, nucleus, cell membrane, and extracellular environment and can be converted to both open (Ca^2+^-bonded cross-linking form) and closed (GTP-bonded signaling form) configurations. Closed configuration has a vital role in BC progression and CSC survival through activation of NFκβ, Akt, and focal adhesion kinase (FAK) signaling (56).

It has been reported that the use of radiation to destroy cancer cells after surgery may convert differentiated cancer cells to CSCs through the expression of CSC markers such as Oct4/Sox2/KLF4. Therefore, in some cancer cases, radiation is not recommended, as it can involve recurrence and metastasis (57). Hypoxia, generated in the depths of the tumor due to lack of oxygen and blood vessels, may regulate the expression of genes involved in CSCs. It may increase the number of CSCs through the conversion of differentiated cancer cells to CSCs (4).

## Signaling Pathways Regulate BCSCs

**Figure 1:**
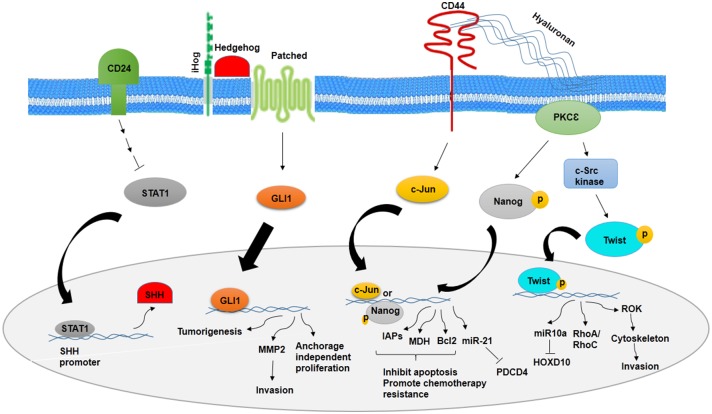
Role of CD24 and CD44 in inhibition and promotion of BCSCs, respectively. CD24 inhibits stemness phenotypes of BCSCs through inhibition of STAT1 and SHH. However, CD44 promotes chemotherapy resistance and invasion of BCSCs through CD44–PKC_ε_-Nanog signaling axis and inducing c-Jun signaling.

It has been noted that a number of signaling pathways including MAP kinase, PI3K/Akt/NFκB, TGF-β, hedgehog (Hh), Notch, Wnt/β-catenin, and Hippo signaling have been implicated in stemness maintenance and regulation of self-renewal, metastasis, and therapeutic resistance into CSCs (12, 14, 56–61). Deregulation of these pathways in normal stem cells may transform them to CSCs. CSCs markers could show a vital role in the regulation of signaling pathways. There is a relationship between CD24 and Sonic hedgehog (SHH), as knocking down CD24 in breast cancer cells have demonstrated increased proliferation, invasion, and tumorigenicity through higher expression of SHH, GLI1, and MMP2. CD24 suppresses the malignant phenotype of BCSCs by downregulating SHH expression through STAT1 inhibition (12) (Figure 1). However, cells with high expression of CD44 show higher expression level of β-catenin and Notch1 and Ki67 (62). CD44-PKC-Nanog signaling axis is involved in BCSCs. Binding of CD44 with protein kinase C_ε_ (PKC_ε_) is mediated by hyaluronan and regulates human breast tumor cells and BCSC functions. Activated PKC_ε_ increases phosphorylation of Nanog, a stem cell marker. Phosphorylated Nanog is translocated into the nucleus and increases miR-21 expression and decreases tumor suppressor program cell death protein 4 (PDCD4) expression. Along with this process, inhibitors of apoptotic proteins (IAPs) and MD11 are upregulated and then leads to antiapoptosis and chemotherapy resistance in BCSCs (63). The other pathway that is mediated by CD44–hyaluronan is HA/CD44-induced c-Jun signaling. Activated c-Jun is translocated into the nucleus and stimulates miR-21 expression, antiapoptosis protein Bcl-2, and IAPs (64). In addition, HA–CD44 interaction promotes c-Src kinase activation and Twist phosphorylation. Phosphorylated Twist is translocated into the nucleus and activates miR-10a expression through binding to Twist binding site(s). This process leads to a decrease in tumor suppressor HOXD10, upregulation of RhoA/RhoC, and stimulation of RhoGTPase-Rho kinase (ROK). ROK activation leads to cytoskeleton activation and breast cancer cell invasion (65) (Figure 1).

In addition, EMT is a process that converts cells with epithelial to mesenchymal phenotypes through decrease in cell–cell adhesion and increase in cell metastasis (40). There are many mediators such as growth factors (epidermal growth factor and TGF-β) that have been involved in several signaling pathways including TGF-β, PI3K/Akt, Wnt, and MAPK and regulate EMT (66). Induction of EMT in normal and cancer cells can generate CSCs with high expression of respective markers, mammosphere formation, colony formation, as well as invasion and metastasis (67). One of the most important levels in the regulation of EMT is the regulation of EMT-inducing transcription factors such as ZEB, Snail, Slug, and Twist. These transcription factors induce EMT through activation of the specific molecular programs in different signaling pathways, which mediate repression of epithelial markers including E-cadherin and induce mesenchymal markers such as vimentin (68, 69). It has been reported that high expression level of ZEB1 has been associated with the activity of several specific molecules, FOXC2, NFκB, SOX2, BCL6, and HIF1α involved in EMT and malignancy of BCSCs (68). Overall, EMT transcription factors contributed to the regulation of signaling pathways in CSCs (66).

### MAPK Signaling Pathway

**Figure 2:**
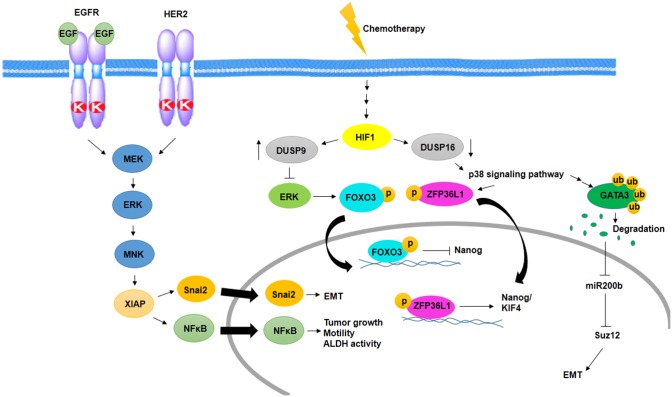
Activation of MAPK signaling pathway in BCSCs. Cooperation between EGFR and HER2 promotes EMT, tumor growth, motility, and ALDH activity through activation of MNK and XIAP in a MAPK-dependent manner. In addition, chemotherapy promotes EMT and induces MAPK signaling pathway and expression of Nanog/KIF4 in a HIF1-dependent manner.

Several studies have shown MAPK signaling activity in aggressive BC and BCSCs. One of the pathways activates MAPK signaling in BCSCs, which is mediated by epidermal growth factor receptor (EGFR) and HER2. In this way, activated ERK stimulates MAPK interacting kinase (MNK) signaling, and then, MNK activates XIAP, its downstream protein. XIAP is an apoptotic inhibitor protein that is upregulated in inflammatory and aggressive BC and links NFκβ signaling activity to MAPK signaling (14). On the other hand, IAP upregulates Snai2 expression, an EMT factor that has an association with increased stem cell properties. Inhibition of IAP protein decreases MAPK activity through downregulation of EGFR and Snai2 expression (70) (Figure 2).

One of the other MAPK activating signaling pathways in BCSC resistance to chemotherapy is mediated by hypoxia-inducible factor-1 (HIF1)-dependent manner. Chemotherapy in an HIF1-dependent manner regulates the expression of dual specificity phosphatase (DUSP9) and DUSP16. Reciprocally, increased expression of DUSP9 inhibits ERK and decreased expression of DUSP16, which activates p38 signaling pathway. ERK inhibition leads to increased expression of Nanog through decreased phosphorylation of FoxO3, and p38 activation stabilizes transcripts of stemness genes such as *Nanog* and *KIF4* via ZFP36L1 phosphorylation, which has been known as a *Nanog* and *KIF4* mRNA binding protein (71) (Figure 2).

p38δ MAPK is involved in cancer cell progression, metastasis, and regulation of BCSC phenotypes as well as EMT. p38δ MAPK-mediated EMT is promoted by the inhibition of miR200b, the downstream of p38γ MAPK. p38γ MAPK inhibits miR200b through induction of GATA3 ubiquitylation and its degradation. Then, expression of a polycomb group protein, Suz12, which is a target of miR200b, promotes EMT (72) (Figure 2).

### PI3K/Akt/NFκB Signaling Pathway

It has been reported that the PI3K/Akt/NFκB axis has been involved in the characterization and drug resistance of BCSCs. Suppressed PI3K/Akt in CSCs overcomes the multidrug resistance (MDR) phenotype through induction of cell apoptosis. The role of PI3K/Akt in BCSCs may be mediated by HER2, which makes tumors more aggressive through unknown mechanism (58). HER2 dysregulation through overexpression of HER2 and its ligand, loss of particular phosphatase, altered dimerization, and decreased receptor turnover lead to an increase in signals and cancer cell proliferation (1). The relevance of HER2 with ALDH1 and phospho-Akt has been shown (1).

On the other hand, inflammation may show a critical role in the relapse of BC through the NFκB–IL-6 pathway, which is involved in BCSC characterization and recurrence of tumor progression. NFκB regulates interleukin-6 (IL-6) and helps in the maintenance of BCSC and conversion of non-CSC to CSC. In addition, some alterations in PI3K/mTOR signaling has been detected in triple negative BC. PI3K/mTOR inhibition through PI3K/mTOR inhibitor or TORC1 inhibitor increases mammosphere formation, a CSC enrichment model, and expression of CSC markers as well as fibroblast growth factor 1 (FGF1) and Notch1 expression in CSCs through increased mitochondrial metabolism (73). Hence, this pathway could be used as a target to make CSCs sensitive to inhibitors of TORC1, which eradicates CSCs. BCSC phenotypes can be mediated by T-box transcription factor 3 (Tbx3) through FGF/Tbx3 signaling pathway. Tbx3 is a member of ancient proteins that is associated with a variety of cancers such as gastric, pancreatic, liver, bladder, and ovary as well as BC. Overexpression of T-box is critical for the maintenance of CSC phenotypes such as self-renewal, drug resistance, and metastasis (74).

It has been reported that steroid hormones are an important player for induction and progression of BC. Despite involvement of hormones and hormone receptors in BC, most studies have reported that BCSCs are hormone receptor negative. It is suggested that ER^+^ to ER^−^ transition is mediated by genetic and epigenetic changes. Proliferation and expansion of ER^−^ CSCs could be driven by ER^+^ BC cells. The secretion of FGF in ER^+^ BC is through ER signaling, by induction of EGF receptor, Notch, and Wnt signaling through FGF paracrine manner in ER^−^ CSCs (75). In addition, progesterone induces proliferation of BCSCs indirectly through NFκB signaling. NFκB signaling activated in PR^+^ cells mediates binding of Receptor activator of NFκB (RANK) with its ligand, RANKL, as a paracrine signal transduction in PR-negative CSCs (75).

### TGF-β Signaling Pathway

The other signaling pathway implicated in BCSCs is TGF-β signaling pathway. TGF-β signaling in BCSCs may be mediated through cytokine receptor signaling indirectly by stimulating expression of family with sequence similarity 3-member C (FAM3C)/interleukin-like EMT inducer (ILEI), an oncogenic protein. Interaction of ILEI with LIFR, its receptor, promotes cytokine receptor signaling through STAT3 activation to drive EMT and metastasis (59). Besides, mTOR signaling, which stabilizes stemness and drug resistance of BCSCs, is mediated by TGF-β. Stable TGF-β exposure promotes proliferation and drug resistance of BCSCs through increased mTOR signaling (76). TGF-β signaling can be regulated by long non-coding RNA (lncRNA), linc-ROR. Linc-ROR is a prognostic and oncogenic factor that is critical in cancer stem cell maintenance. Overexpression of linc-ROR upregulates TGF-β signaling, and then, TGF-β signaling promotes proliferation and invasion of BCSCs (77). Activated Smad and non-Smad through TGF-β signaling pathway leads to the induction hyaluronan synthase 2 (*Has2*) and *Has2* antisense (*HAS2-AS*) transcripts, high mobility group AT-Hook 2 (*Hmga2*), and EMT inducing transcription factors such as Snail, Snai1, Hmga2, and fibronectin 1 (78). In addition, induction of *Hmga2, Has2-AS*, and Has2 are accompanied by activation of Akt and Erk1/2 MAPK signaling. These factors have been contributed to cancer stem cell maintenance, migration, and EMT of BCSCs (78).

### Wnt/β-Catenin and Notch Signaling Pathway

Wnt and Notch signaling pathways are other signaling pathways involved in cancer stem cell maintenance (60, 61). Wnt and Notch signaling pathways may be activated by HIF-2α overexpression, which is mediated by hypoxia. HIF-2α overexpression promotes stem cell phenotype conversion, drug resistance of BCSCs, and overexpression of BCSC markers (79). Among several factors that have been involved in Wnt signaling, overexpression of glycogen synthase kinase 3 beta (GSK3β) correlates with poor patient survival. Overexpression of GSK3β induces EMT and cancer stem cell properties in BCSCs (80). The contribution of Wnt signaling in stemness phenotypes of CSCs is mediated by programmed death 1 (PD-1), which is overexpressed in BCSCs. In addition, Wnt activators and Wnt inhibitors up- and down-regulate PD-1, respectively. High expression of PD-1 is associated with stem cell features (81). Recently, it has been reported that kinesin family member 11 (KIF11), a motor protein essential in mitosis, contributed to the regulation of Wnt/β-catenin in BCSCs. It increases stemness phenotypes of BCSCs through activation of Wnt/β-catenin (82). The other protein that regulates Wnt/β-catenin in BCSCs is Nectin-4. Nectin-4, a junction protein, is well-known as a BCSC marker and is implicated in metastasis of BC and BCSCs. Overexpression of Nectin-4 upregulates Wnt/β-catenin signaling pathway, which is implicated in EMT, metastasis, and proliferation of BCSCs (83). Wnt/β-catenin signaling regulates EMT by induction of ZEB1 expression through binding β-catenin to ZEB1 promoter. In addition, ZEB1 promoter has two STAT3 binding sites, suggesting strong correlation between ZEB1 and STAT3 (68). In addition, cytokeratin 5, which is enriched in BCSCs, alters the function of β-catenin in two ways. Knockdown of cytokeratin 5 interrupts the transcription activity of β-catenin, even in response to Wnt stimuli. On the other hand, cytokeratin 5 induced by Wnt stimuli promotes deficiency of β-catenin and the other junction protein of cell membrane such as E-cadherin. It suggests dual function of cytokeratin 5 in relation to β-catenin in the cell membrane of BCSCs (84).

It has been shown that there is an association between estrogen signaling and Notch signaling in ERα+ BC cells, which is mediated by Delta-like protein 1 (DLL1), Notch ligand. Upregulation of Notch signaling by overexpression of DLL1 promotes proliferation, migration, angiogenesis, and cancer stem cell phenotypes in ERα+ BC cells. This function for DLL1 is specific in ERα+ BC cells. Thus, it suggests that estrogen and estrogen signaling stabilizes DLL1 through inhibition of ubiquitylation and degradation of DLL1 (85).

**Figure 3:**
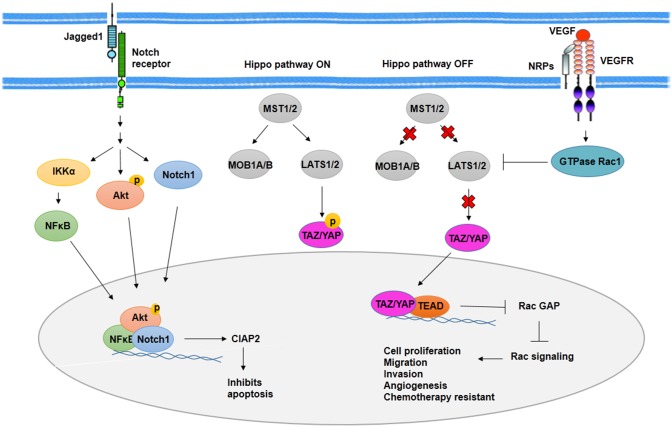
Notch signaling activity and Hippo pathway in BCSCs. As transcription factors, Notch1, phosphorylated Akt, and NFκB induce expression of CIAP2 and inhibit apoptosis through Notch signaling activity in BCSCs. On the other hand, Hippo pathway promotes cell proliferation, migration, invasion, angiogenesis, and chemotherapy resistance in BCSCs through translocation of YAP/YAZ into the nucleus and activation of Rac signaling. In addition, VEGFR/NRPs promotes stemness phenotypes of BCSCs, indirectly through inhibition of Hippo pathway.

In BCSCs, activated Notch signaling by jagged1 leads to Akt phosphorylation and IκB kinase α (IKKα), NFκB, and Notch1 activation. Recruitment of IKKα, NFκB, and Notch1 to antiapoptotic gene cIAP-2 promoter increases expression of cIAP-2 (86) (Figure 3). Along with Notch1, activated Notch3 has contributed to the maintenance of BCSCs properties. It is mediated by overexpression of cartilage oligomeric matrix protein (COMP) in BCSCs. COMP increases interaction of Notch3 and jagged1, which leads to overactivation of Notch3 signaling pathway (87).

### Hedgehog Signaling Pathway

One of the signaling pathways upregulated in aggressive subtype of breast cancer is the hedgehog signaling pathway (61). There is an association between overexpression of hedgehog signaling pathway regulators such as SHH, DHH, IHH, PTCH1, SMO, and GLI1 with proliferation, migration, and aggressiveness of BC (88). It has been observed that SHH/GLI1^+^ samples express high levels of vimentin and Snail. It suggests that the hedgehog signaling pathway and SHH/GLI1 axis are involved in EMT, migration, and invasion of BCSCs (89).

### Hippo Signaling Pathway

Among signaling pathways involved in CSCs, the Hippo pathway plays an important role in organogenesis and regeneration. Molecules that regulate Hippo signaling included regulatory kinases such as MST1, MST2, LATS1, LATS2, SAV1, MOB kinase 1A, MOB kinase 1B, and transcriptional coactivators such as TAZ and YAP. Alteration in Hippo signaling through deregulation of these molecules leads to increased cell proliferation and tissue overgrowth in mammary. Phosphorylated TAZ and YAP mediated by activation of kinase cascade suppresses the inclusion of TAZ and YAP into the nucleus as a result of the overexpression of TAZ and YAP and deregulation of kinases. The inclusion of TAZ and YAP into the nucleus leads to transcription of TAZ and YAP target genes involved in cell proliferation, migration, and invasion as well as resistance to chemotherapy (Figure 3). Overexpression of TAZ/YAP has been reported in BCSCs to lead to increased activity of MDR protein and cooperation between TAZ and ECM (90).

### Toll-Like Receptor Activity

Toll-like receptors (TLRs) are a class of proteins implicated in inflammatory signaling and tumorigenesis (91). TLRs are overexpressed in a variety of cancers such as esophageal, gastric, as well as BC (91). Among TLRs, TLR2, and TLR3 are overexpressed in BCSCs. TLR2 and its ligand, high-mobility-group box 1 (HMGB1), display a key role in BCSC phenotypes such as self-renewal and metastatic and tumorigenic activity through inducing TGF-β and IL-6 secretion, Smad3 and STAT3 activation, and IκBα phosphorylation (92). In addition, activation of TLR3 promotes BC cells to BCSC phenotypes. Activation of TLR3 leads to coactivation of β-catenin and NFκB signaling pathways essential for increased BCSC phenotypes (91).

## Chemotherapy and Radiotherapy Resistance in BCSCs

BCSCs are the main cause of chemotherapy and radiotherapy resistance of BC. These cells may up- or down-regulated essential genes and then initiate signaling pathways, which make tumors more aggressive than before and resistant to therapy. Several mechanisms and molecules have been proposed in therapeutic resistance of BCSCs. There are two main mechanisms: pump and non-pump resistance. Non-pump resistance is related to stimulation of BCL2 protein, an antiapoptotic protein, whereas pump resistance is related to overexpression of ABCG2. ABCG2 can flush out the chemotherapy drugs, which dilutes the concentration of intracellular drug (93). It has been reported that Survivin, an inhibitor of apoptosis, is overexpressed in BC cells as well as BCSCs. Survivin overexpression is related to reduced apoptosis and increased drug resistance. Downregulation of Survivin with anticancer agents such as prodigiosin may make BCSCs more sensitive to chemotherapeutic agents (94). Furthermore, ALDH is contributed to BCSC therapy resistance through direct and indirect manners. In direct manner, ALDH removes oxygen radicals that are produced by chemo- and radiotherapy, but in indirect situation, it produces nicotinamide adenine dinucleotide phosphate (NADP), an antioxidant agent (95). In addition, one of the specific mechanisms of BCSCs that protects them against reactive oxygen species (ROS) production by radiotherapy is using an ROS scavenging factor. ROS scavenging is mediated by the upregulation of genes such as catalase (*CAT*), superoxide dismutase (*SOD*), and glutathione peroxidase (*GPx*) in BCSCs (95).

Various studies have reported other important molecules involved in chemotherapy and radiotherapy resistance of BCSCs. Caveolin-1 is involved in chemoresistance of BC, its expression which is accompanied by coexpression of β-catenin and ABCG2. Caveolin-1 is upregulated after BC chemotherapy in BCSCs. Caveolin-1 promotes β-catenin/ABCG2 signaling via GSK3β suppression and Akt activation (13). In addition, protein kinase D1 (PRKD1) has been identified as a critical modulator of stemness phenotypes and therapy resistance of BCSCs within GSK3/β-catenin signaling. PRKD1 inhibitor and miR-34a, which target PRKD1 through binding to the PRKD1 3′-untranslated region (UTR), suppress tumor growth and proliferation as well as drug resistance and induce apoptosis (96). Cells with stem-like phenotypes such as having ALDH activity and drug resistance exhibit high mitochondrial mass (97). It proposes that chemo-resistance ability of BCSCs is driven by high function of mitochondria (97).

Recent studies suggested that the level of cholesterol may limit the efficiency of radiotherapy in cancer patients. There is a relationship between plasma levels of lipoproteins and radio-resistance in inflammatory BC and BCSCs. Transporters of cholesterol (lipoproteins) such as high-density lipoprotein (HDL), low-density lipoprotein (LDL), and very-low-density lipoprotein (VLDL) may regulate radiotherapy sensitivity. Decreasing cellular cholesterol through treatment with cholesterol-lowering agents such as HDL decreases radiotherapy resistance, whereas VLDL decreases radiotherapy sensitivity (98).

**Figure 4:**
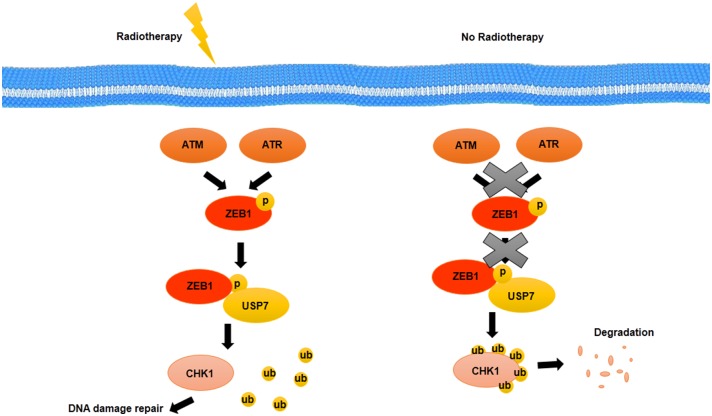
Activation of repair mechanisms due to radiotherapy induced-damages in BCSCs. DNA damage induced by radiotherapy is repaired through an ATM–ZEB1–CHK1 signaling axis. Activated ATM and ATR kinases phosphorylate ZEB1. ZEB1 interacts with USP7, a deubiquitylating enzyme, and promotes the deubiquitylation ability of USP7 that stabilizes CHK1.

EGFR and HER2, two growth factor receptors that have been overexpressed in BCSCs, have an important role in radiotherapy resistance of BCSCs. EGFR and HER2 inhibitors such as GW572016 may make BCSCs sensitive to radiotherapy as well as inhibit proliferation through inhibition of receptor phosphorylation (99). The therapeutic resistance that is modulated by EMT has been detected in various types of cancer. Activation of genes related to EMT such as Snail and Slug accompanied with activation of Notch signaling pathway suppressed the expression of genes related to p53-mediated apoptosis (100). One of the other prosurvival factor that is involved in EMT and is induced through radiation in HER2+ BCSCs is NFκB (100). NFκB induces expression of HER2. HER2, as a receptor, activates STAT3, which upregulates stemness markers. It suggests that there is a relationship between EMT, cell stemness, and radioresistance of BCSCs (100). Non-CSCs show more level of apoptosis during radiotherapy, whereas CSCs confront with radiotherapy-induced damages through activation of repair mechanisms (101). Activation of repair mechanisms due to radiotherapy-induced damages is mediated by zinc finger E-box binding homeobox 1 (ZEB1). ZEB1, as a transcription factor, not only has been implicated in embryonic development through EMT induction but also may promote therapy resistance as well as metastasis and invasion in BCSCs. The role of ZEB1 in radiotherapy resistance is modulated by ATM and ATM and RAD3-related kinase (ATR) kinases. Genetic deficiencies and DNA variations that are caused by radiotherapy will be repaired through an ATM–ZEB1–CHK1 signaling axis activation. Activated ATM and ATR kinases through radiotherapy-induced damages phosphorylate ZEB1. ZEB1 interacts with deubiquitinase, ubiquitin-specific peptidase 7 (USP7) and promotes the deubiquitination ability of USP7 and then stabilizes CHK1 (102) (Figure 4). Thus, targeting ZEB1 by inhibitors such as miR-205 and miR-200c as radiosensitizer may be used as strategies for confronting with radio-resistance of BCSCs (102). In addition, ZEB1 is contributed to non-CSC conversion to CSCs in basal type of BC by inducing TGF-β, a microenvironment stimuli of basal BC (102).

## Metastasis in BCSCs

Metastasis has been described by two potential ability of BCSCs, migration, and invasion. As mentioned above, not only EMT is an important process in chemotherapy and radiotherapy resistance but also it is implicated in metastasis of BCSCs. Snail, a transcriptional repressor, is one of the most important inducer of EMT through repression of E-cadherin expression. In addition, the expression of snail maintains malignancy phenotypes of BCSCs such as metastasis. Therefore, it could be suggested that snail is a potential target to prevent metastasis of BCSCs (40). Along with snail, ZEB1, an EMT inducer, promotes metastasis and tumor progression in BCSCs through repression of E-cadherin expression as well as epithelial polarity factors such as HUGL2, Crumbs3, and Pals1-associated tight junction (PATJ) expression. This leads to adhesion reduction and tumor cell dissociation and finally increased invasion (102). Additionally, deubiquitinating enzymes (DUBs), especially USP37 and USP2, are involved in EMT, metastasis, as well as chemotherapy resistance of BC and BCSCs. USP37 and USP2 are overexpressed in metastatic BC and BCSCs. USP37 mediates hedgehog signaling pathway through stabilizing components such as Smo and GLI-1. Finally, hedgehog signaling drives to stemness, invasion, and EMT of BCSCs (103). In addition, USP2 activates Bmi1 and EMT by stabilizing Twist through removing ubiquitylation mediated by beta-transducin repeats-containing proteins (β-TrCP), a cellular E3 ubiquitin ligase (104).

Extracellular matrix protein 1 (ECM1) is an important prognosis marker for the invasion of breast cancer as well as migration and drug resistance. In addition, ECM1 controls the expression of genes involved in EMT and BCSC maintenance through induction of Mucin1 (MUC1). The cytoplasmic tail of MUC1 has a physical association with β-catenin that stabilizes and increases posttranslational expression of β-catenin. Therefore, altered expression of β-catenin changes the expression of genes related to BCSC phenotypes such as invasion and EMT progression (105).

CD36, a member of the scavenger receptor family with binding to its receptor, thrombospondin-1 (TSP-1) plays an important role in metastasis of BCSCs through several pathways. CD36 binds to TSP-1 and TGF-β1 and promotes proliferation and migration of BCSCs. On the other hand, it can interact with NFκB and STAT3, which express genes related to carcinogenesis. In addition, CD36 has a main role in BCSC metastasis through uptaking fatty acids and regulating 3-hydroxy-3-methylglutaryl-CoA synthase 2 (HMGCS2) enzyme and β-oxidation of fatty acids (74). Furthermore, induction of NFκB signaling pathway as a result of phosphodiesterase 3A (PDE3A)-suppressed cAMP/PKA promotes expression of OCT4 as well as translocation of CCDC88A, which boots invasion and metastasis of BCSCs (106).

The role of CD44 in cancer progression, invasion, metastasis, and angiogenesis of BCSCs is remarkable. There are several ligands such as hyaluronic acid, osteopontin, and matrix metalloprotease that bond to CD44. Binding ligands to CD44 induces cleavage of CD44 at extracellular and transmembrane domain sites by membrane-type 1 matrix metalloprotease (MT1-MMP) and presenilin-1/g secretase, respectively. Then, the intracellular domain fragment of CD44 (CD44-ICD) displaces into the nucleus and activates expression of genes related to migration, invasion, metastasis, and angiogenesis of BCSCs (107).

## Angiogenesis in BCSCs

Cell survival mediated by hypoxic condition after radiotherapy is contributed to the angiogenesis of CSCs (101). In both hypoxic and normoxic conditions, CSCs show high expression of vascular endothelial growth factor (VEGF). In addition, HIF-1, which is stabilized in hypoxic condition, regulates expression of VEGF (101). VEGF signaling has been contributed to proliferation, drug resistance, and cancer stem cell phenotypes as well as angiogenesis (108). Activated VEGF signaling reveals to be responsible for activation of GTPase Rac1, which then inhibits Hippo kinase LATS. The inhibition of Hippo kinase LATS leads to the activation of Hippo effector TAZ and YAP. TAZ and YAP, in a complex with transcription factor TEAD, repress expression of Rac GTPase-activating protein (Rac GAP) and then activate Rac signaling pathway, which promotes proliferation, angiogenesis, and the other cancer stem cell phenotypes (108) (Figure 3). In addition, VEGF-A/neuropilin-1 (NRP-1) axis is contributed to stemness properties such as angiogenesis through activating Wnt/β-catenin pathway.

The role of CD44 has been confirmed in angiogenesis as well as metastasis. CD44 binds to its ligand, hyaluronic acid, as well as VEGF through its binding domains and increases angiogenesis (107).

## miRNAs Upregulated in BCSCs

miRNAs are introduced as one of the non-coding RNAs classes that suppress gene expression through inhibition of translation. Several studies suggest that several miRNAs regulate stemness phenotypes of BCSCs through targeting genes involved in signaling pathways. Table 1 presents miRNAs that are upregulated in BCSCs and are involved in BCSC maintenance and phenotypes.

## BCSCs and Therapy Strategies

Substantial shreds of evidence support that resistance of BCSCs to chemotherapy and radiotherapy have been associated with metastasis and relapse of BC after treatment (109). Therefore, discovering potential strategies and targeting BCSCs could be effective to overcome the relapse of BC. Recently, various strategies such as herbal medicine, biological agents, anti-inflammatory drugs and monoclonal antibodies, nanoparticles, and microRNAs have been studied for targeting BCSCs. Signaling pathways that are dysregulated in BCSCs are the best candidate for developing novel strategies for targeting BCSCs. In addition to signaling pathway inhibitors such as Notch and γ-secretase inhibitor (MK-0752), hedgehog inhibitor (Vismodegib, GDC-0449), Wnt signaling pathway inhibitor (PKF118-310 and pyrvinium pamoate), PI3K/Akt/mTOR pathway inhibitor (Everolimus, RAD001), CXCR2 inhibitor (Reparixin), and EGFR/HER2 inhibitors (Lapatinib and Herceptin), which target signaling pathways directly (110), offer several breast cancer treatment strategies via targeting BCSCs.

### Herbal Medicine

Recently, several studies have implicated natural components and herbal medicine in BC treatment by targeting BCSCs (111–113). These components have several advantages such as fewer side effects in comparison with non-natural drugs. Recently, it has been shown that *Viola odorata* extract, a herbal medicine, decreases stemness potency and cell viability of BC-cell-derived mammospheres as well as induction of apoptosis (114). It has been reported that a natural component of brown algae, phloroglucinol (PG), suppresses BCSC proliferation through reduction of Oct4, Sox2, β-catenin, and CD44 expression. In addition, it inhibits KRAS, RAF1/ERK, and PI3K/Akt signaling pathways in BCSCs (109). Capsaicin, an active ingredient of chili peppers, has shown anticancer and antiproliferation activity on BCSCs through inhibition of translocation Notch intracellular membrane domain (NICD) into the nucleus and inhibition of Notch signaling (115). In addition, Psoralidin (Pso), a small herbal molecule effectively inhibits Notch1 signaling, which leads to reduction in growth, migration, and invasion of BCSCs through downregulation of vimentin and β-catenin and upregulation of E-cadherin expression (116).

Nobiletin, a flavonoid derived from citrus peel, has anticancerous activity by binding to CD36 and suppressing CD36/STAT3/κ-κB signaling axis, which inhibits angiogenesis, migration, and metastasis of BCSCs (74). Apigenin, the other flavonoid detected in many herbal medicine, suppresses stemness properties of BC cells by inhibiting YAP/TAZ activity through disrupting YAP/TAZ-TEADs complex (79).

5,6,7-Trihydroxy-2-phenyl-4H-1-benzopyran-4-one (Baicalein), an active component of *Scutellaria baicalensis Georgi*, targets BCSCs and suppresses stemness potential of BCSCs by induction of apoptosis through inducing ROS and changing mitochondrial membrane potentials as well as downregulating Wnt/β-catenin pathway (117).

### Biological Agents

Implicating biological agents against BCSCs have been observed in several researches. FK506-binding protein-like (FKBPL) and AD-01 (one of the its derivatives) are novel biological agents that have an effective role in inhibiting tumor growth initiation, angiogenesis, and metastasis of BC cells as well as BCSCs through targeting CD44 pathway and downregulating DLL4, a Notch ligand, and Notch4 (118, 119). Disabling mitochondria in BCSCs could be introduced as an effective way of eradicating BCSCs. Mitocans are a group of components that target mitochondria and induce apoptosis in BCSCs. They could be suggested as anticancer agents. Mito Vitamin E succinate (MitoVES) is a component of mitocans that induces apoptosis in BCSCs through targeting mitochondria complex II (120). Targeting PI3K/Akt signaling pathway, as an important pathway in MDR of BCSCs, has been suggested as an antineoplastic strategy to overcome the MDR phenotype of BCSCs. PI3K inhibitors such as BKM120 inhibit PI3K/Akt signaling, which leads to activation of apoptotic caspases such as caspase-3/7 and caspase-9 (58). In addition, a novel chemical component, 1-ferrocenyl-3-(4-methylsulfonylphenyl)propen-1-one (FMSP), targets several signaling pathways such as Wnt/β-catenin and Notch through downregulating Wnt1, Notch1, β-catenin, SOX2, CXCR4, and ALDH1A1 as well as apoptosis induction through ROS production (121). In addition, 5-aza-2′-deoxycytidine (DAC), an epigenetic drug, induces differentiation, S phase cell cycle arrest through induced expression of *p53, p21, p16, p15, BRCA1*, and *BRCA2*, and reduced expression of *ABCG2* through demethylation mechanism (122).

### Anti-inflammatory Agents and Monoclonal Antibodies

Developing anti-inflammatory drugs and monoclonal antibodies could be useful for preventing proliferation, metastasis, and drug resistance of BCSCs. Aspirin (ASA) prodrug is one of the anti-inflammatory drugs that has shown antineoplastic activity without toxicity through targeting NFκB–IL6 pathway and cyclooxygenase (COX)/prostaglandin in BCSCs (123, 124). In addition, ASA blocks angiogenic factors that are secreted by CSCs and contributed to tumor angiogenesis. It blocks paracrine–autocrine signaling between endothelial cells and CSCs (125).

Previously, it has been shown that EGFR is a critical player in tumor initiation, metastasis, and drug resistance of BC and BCSCs. Therefore, implication of EGFR monoclonal antibody such as Cetuximab effectively could be suggested as a useful strategy to reduce BCSC population (126). Bevacizumab, an anti-VEGF antibody targets VEGF and suppresses angiogenesis of BCSCs (127). Recently, BCSC markers such as CD44 and EpCAM have been used for developing therapeutically monoclonal antibodies. For example, MT110, anti-EpCAM and P245, and anti-CD44 are potential therapeutic monoclonal antibodies that have exhibited anticancer activity against BCSCs (128).

### Nanoparticles

Recently, nanoparticles have been an interesting approach for targeting cancer cells especially CSCs. Specific phenotypes of CSCs can be used for designing nanoparticles. Among nanomaterials, mesoporous silica particles have demonstrated efficient drug delivery in cancer therapy. It has been designed based on glycolytic phenotype and Notch activity of CSCs. Uptaking mesoporous silica nanoparticles accompanied with γ-secretase inhibitor by BCSCs targets Notch signaling pathway and eliminates stemness properties of BCSCs.

### MicroRNAs

miRNAs are the most attractive topics in BCSCs. They have been implicated in targeting BCSCs through several signaling pathways. Table 2 presents tumor-suppressing miRNAs that are downregulated in BCSCs and are implicated for targeting BCSCs as a novel strategy with their suggested functional pathways.

## Conclusion

BCSCs are one of the main reasons for tumor development and recurrence of BC after treatment. These cells have some specific markers: CD44^+^/CD24^−^, ALDH activity, and ESA or CD326 (EpCAM), CD133, CD166, Lgr5, CD47, and ABCG2 in different types of BCs. BC cells' conversion to BCSCs due to genetic and epigenetic changes leads to deregulation of several signal transduction pathways such as MAP kinase, PI3K/Akt/NFκB, TGF-β, Wnt/β-catenin, Notch, hedgehog, and Hippo signaling. Deregulation of these signaling pathways and other factors leads to several stemness phenotypes such as chemotherapy and radiotherapy resistance, EMT, invasion, migration, and angiogenesis. Additionally, the role of miRNAs in stemness features of BCSCs is remarkable. A variety of miRNAs upregulated in BCSCs are contributed to stemness phenotypes of BCSCs. Recently, implicating novel treatment strategies such as herbal medicine, biological agents, anti-inflammatory drugs, monoclonal antibodies, nanoparticles, and microRNAs that target different signaling pathways and other deregulated genes directly or indirectly could be effective for targeting BCSCs.

## Author Contributions

SY and SS: conception, providing the data and design, manuscript writing. FS, MN, AG, and KG: conception and final approval of manuscript.

## Conflict of Interest

The authors declare that the research was conducted in the absence of any commercial or financial relationships that could be construed as a potential conflict of interest.

## Abbreviations

ADAM17: ADAM metallopeptidase domain 17
ALDH: aldehyde dehydrogenase
ASA: aspirin
ATM: ataxia telangiectasia mutated
ATR: ATM- and RAD3-related
DAC: 5-aza-2′-deoxycytidine
β-TrCP: beta-transducin repeat-containing proteins
BC: breast cancer
BCSCs: breast cancer stem cells
CSCs: cancer stem cells
COMP: cartilage oligomeric matrix protein
CAT: catalase
COX: cyclooxygenase
DLL: delta like protein
DUBs: deubiquitinating enzymes
DUSP9/DUSP16: dual specificity phosphatase
EpICD: EpCAM intracellular domain
EGFR: epidermal growth factor receptor
EMT: epithelial–mesenchymal transition
ESA: epithelial specific antigen
ER: estrogen receptor
ECM1: extracellular matrix protein 1
FAM3C: family with sequence similarity 3 member C
FMSP: 1-ferrocenyl-3-(4-methylsulfonylphenyl)propen-1-one
PDE3A: phosphodiesterase 3A
FGF1: fibroblast growth factor 1
FKBPL: FK506-binding protein like
FAK: focal adhesion kinase
FHL2: four and a half LIM domains 2
GPx: glutathione peroxidase
GSK3β: glycogen synthase kinase 3 beta
Hh: hedgehog
HDL: high-density lipoproteins
Hmga2: high mobility group AT-Hook 2
HMGB1: high-mobility-group box 1
HER2: human epidermal growth factor receptor 2
Has2: hyaluronan synthase 2
HAS2-AS: hyaluronan synthase 2-antisense
HA: hyaluronic acid
HMGCS2: 3-hydroxy-3-methylglutaryl-CoA synthase 2
HIF1: hypoxia-inducible factor-1
IKKα: IκB kinase α
IAPs: inhibitors of apoptosis proteins
IL-6: interleukin-6
ILEI: interleukin-like EMT inducer
CD44-ICD: intracellular domain fragment of CD44
KIF11: kinesin family member 11
LncRNA: long non-coding RNA
LDL: low-density lipoproteins
MNK: MAPK interacting kinase
MMP: matrix metalloprotease
MT1-MMP: membrane type 1-matrix metalloprotease
MitoVES: Mito Vitamin E succinate
MUC1: mucin1
MDR: multidrug resistance
NRPs: neuropilins
NADP: nicotinamide adenine dinucleotide phosphate
NICD: notch intracellular membrane domain
OPN: osteopontin
PATJ: Pals1-associated tight junction
PG: phloroglucinol
PSEN2: presenilin-2
PR: progesterone receptor
PDCD4: program cell death protein 4
PD-1: programmed death 1
PKC_ε_: protein kinase C_ε_
PRKD1: protein kinase D1
Pso: psoralidin
Rac GAP: Rac GTPase-activating protein
RANKL: RANK ligand
ROS: reactive oxygen species
RANK: receptor activator of NFκB
RTK: receptor tyrosine kinase
ROK: rho kinase
SHH: Sonic hedgehog
SOD: superoxide dismutase
Tbx3: T-box transcription factor 3
TSP-1: thrombospondin-1
TLRs: Toll-like receptors
TGF-β: Transforming growth factor beta
TG2: transglutaminase
TNBC: triple negative breast cancer
USP: ubiquitin specific peptidase
VEGF: vascular endothelial growth factor
VLDL: very-low-density lipoproteins
ZEB1: zinc finger E-box binding homeobox 1.
